# Supplementary material for: Splice-Junction-Based Mapping of Alternative Isoforms in the Human Proteome
Source: Cell Rep. Author manuscript; Available in PMC 2020 Jan 15. (PMC6961840; doi:10.1016/j.celrep.2019.11.026)
Supplement: 2 [file NIHMS1546469-supplement-2.zip › DF1/PXD010154/AdrenalGland/AdrenalGland_24_ASB8_EYLLLLSR.pdf]

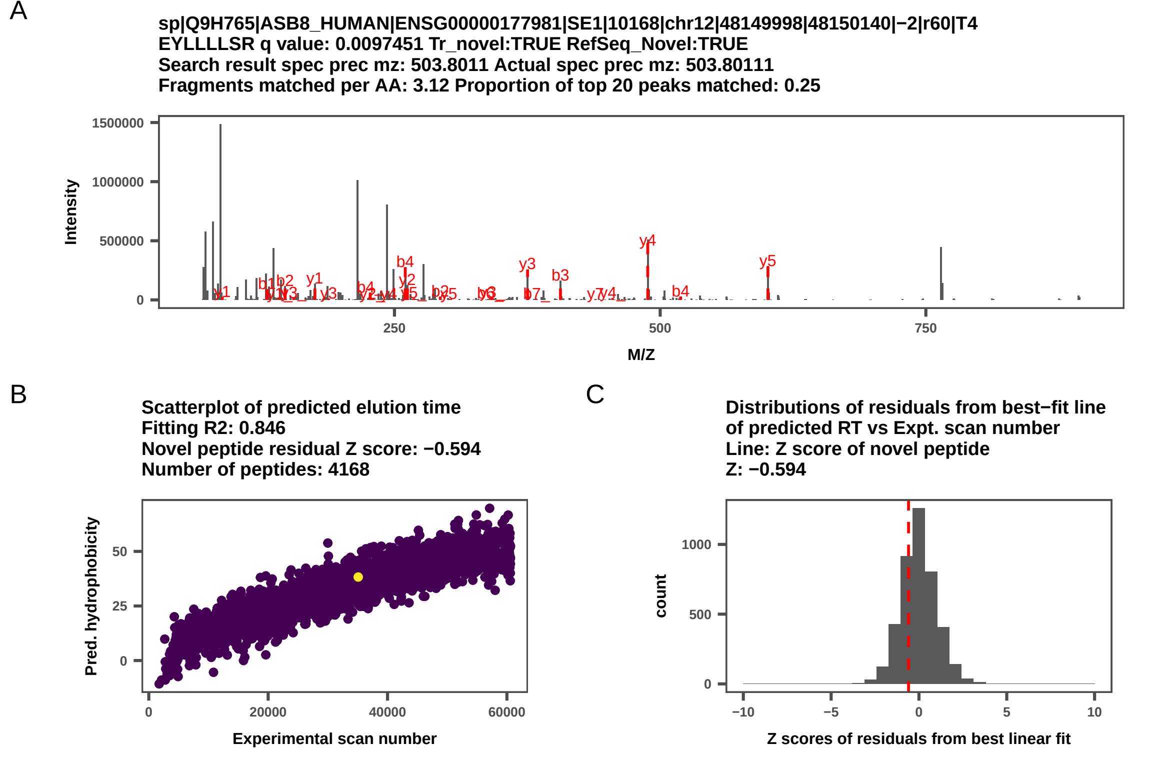
A
sp|Q9H765|ASB8_HUMAN|ENSG00000177981|SE1|10168|chr12|48149998|48150140|−2|r60|T4
EYLLLLSR q value: 0.0097451 Tr_novel:TRUE RefSeq_Novel:TRUE
Search result spec prec mz: 503.8011 Actual spec prec mz: 503.80111
Fragments matched per AA: 3.12 Proportion of top 20 peaks matched: 0.25
Intensity
1500000
1000000
500000
0
M/Z
250
500
750
y4
y5
b4
y3
b3
B
Scatterplot of predicted elution time
Fitting R2: 0.846
Novel peptide residual Z score: −0.594
Number of peptides: 4168
Pred. hydrophobicity
50
25
0
Experimental scan number
0
20000
40000
60000
C
Distributions of residuals from best−fit line
of predicted RT vs Expt. scan number
Line: Z score of novel peptide
Z: −0.594
count
1000
500
0
Z scores of residuals from best linear fit
−10
−5
0
5
10
